# Prevalence of tinea pedis among adults in primary health care settings in Qatar: A cross-sectional study

**DOI:** 10.5339/qmj.2025.46

**Published:** 2025-06-09

**Authors:** Hani Abdalla, Menatella Abdelnaby, Nadeen Khamis, Fatima AlYafei, Suad Alahwal, Ahmed S. Alnuami

**Affiliations:** 1 Primary Health Care Corporation, Doha, Qatar; 2 Qatar University, Doha, Qatar; 3 Hamad Medical Corporation, Doha, Qatar *Email: haabdalla@phcc.gov.qa

**Keywords:** Tinea pedis, athlete’s foot, prevalence, risk factors, diabetes mellitus, Qatar

## Abstract

**Background:**

Tinea pedis, commonly known as athlete’s foot, is a fungal infection of the feet, particularly affecting the space between toes, and it is easy to treat. While it is prevalent worldwide, limited data exist on its prevalence in Qatar. This study aims to determine the prevalence of tinea pedis and identify its associated risk factors among adults in the primary healthcare settings in Qatar.

**Methods:**

A cross-sectional study analyzed electronic medical records (EMRs) of adults aged ≥18 years who registered with the Primary Health Care Corporation (PHCC) between July 2018 and June 2023. A total of 1,002,594 EMRs were studied, and statistical analysis was performed using the Statistical Package for Social Sciences (SPSS) version 28. We obtained approval with reference number BUHOOTH-D-23-00039 to conduct this study from the Institutional Review Board (IRB) at PHCC.

**Results:**

The overall prevalence of tinea pedis was 1.8%. The risk of tinea pedis increased significantly with age, being 25.1 times higher among individuals aged 70 years or older compared to younger adults (18–29 years). Males were 1.6 times more likely to be affected than females. North Africans had a 3.9 times increased risk. Obesity was a major risk factor, with morbidly obese individuals being 15.1 times more likely to develop tinea pedis than underweight individuals. Diabetics had a 7.1-fold increased risk compared to non-diabetics. HbA1c% values of <7, 7.0–7.9, and ≥8 were considered as recommended control, less-stringent control, and poor control of diabetes mellitus, respectively. Poor control of diabetes elevated the risk by 20% compared to recommended control. All associations were statistically significant.

**Conclusion:**

Tinea pedis is a significant health issue in Qatar, particularly among older adults, males, obese individuals, diabetics, and those with poorly controlled diabetes. These findings can increase awareness among local physicians about the need to screen high-risk groups and promote prevention of modifiable risk factors through targeted interventions and patient education.

## Introduction

Tinea pedis, also known as athlete’s foot, is an infection caused by a dermatophyte fungus. It can affect any portion of the foot but is most often found in the space between the toes. It is spread through contact with infected skin scales or contact with fungi in damp areas.^1^ While often considered a minor disease, tinea pedis can lead to more serious complications if left untreated. Notably, it may cause severe bacterial cellulitis, a deeper skin infection that can result in swelling, redness, and pain.^2^ Tinea pedis is generally responsive to treatment. Topical antifungal medications, such as clotrimazole or terbinafine, are commonly effective. Treatment duration varies based on the severity of the infection, but typically ranges from 1 to 6 weeks.^3^

The worldwide prevalence of tinea pedis was estimated at 3%.^3^ A study done in Spain showed a prevalence of tinea pedis of 2.9%.^4^ In our region, a national survey in Saudi Arabia showed tinea pedis prevalence of 15.5% among diabetic patients,^5^ while another study conducted in Egypt revealed a higher figure of 42.6% among diabetics.^6^ A study from Iraq found that 24.8% of patients with tinea pedis were diabetics.^7^ A nationwide study conducted in Korea examined the risk factors among Korean adults and highlighted a significantly increased risk in individuals with elevated waist circumference or high BMI, male gender, older age group, heavy drinking, engaging in mild-to-heavy exercise, and those with diabetes.^8^

A structured literature search revealed that no study has been conducted specifically about the prevalence of tinea pedis in Qatar. The only research article published in Qatar in 2015 that investigated the burden of serious fungal infections did not specifically report on tinea pedis.^9^ Therefore, we aim to direct the focus of the current study to both the current prevalence of tinea pedis and the associated risk factors in the general and diabetic populations of Qatar. Determining the prevalence and burden of tinea pedis will raise awareness among the community and physicians. Examining risk factors associated with tinea pedis will enable us to focus on at-risk individuals, conduct regular screenings, and advise them on preventive measures by maintaining proper foot hygiene, keeping feet dry, and avoiding barefoot in communal areas or swimming pools.^10^

## Methods

### Study design and setting

A cross-sectional study analyzed data extracted anonymously from electronic medical records (EMR) of individuals registered with the Primary Health Care Corporation (PHCC) in Qatar. PHCC is the principal publicly funded provider of primary healthcare services in Qatar, operating a network of 31 health centers. Primary healthcare centers serve all three major regions of the country: central, north, and west, ensuring coverage and facilitating equitable access to primary healthcare services for the entire population.^11^ Access to PHCC services is available to residents of Qatar, including citizens and expatriates. PHCC has approximately 1.78 million active registered persons (60% of Qatar’s population), indicating that a significant portion of Qatar’s population utilizes PHCC services.^12^ PHCC offers a wide spectrum of services, including preventive and curative care, as well as supporting clinical research. PHCC plays a major role in Qatar’s National Health Strategy 2024–2030, which focuses on three key priority areas: enhancing population health and well-being, delivering excellence in patient care and service, and strengthening the efficiency and resilience of the health system.^13^

### Study population

Adults aged 18 years and older who were registered with PHCC between July 2018 and June 2023.

### Outcome measures

The primary outcome measure was the prevalence of tinea pedis among the study population.

### Explanatory variables

The list of independent (explanatory) variables included age, gender, nationality, body mass index (BMI), diagnosis of diabetes mellitus, and HbA1c% blood sugar test as a measure of diabetes control.

### Data extraction

Data were extracted from the EMR by the Department of Business Health Information and validated by testing the codes and filters used for data extraction. Data included age, gender, nationality, BMI, presence of diabetes mellitus, hemoglobin A1c (HbA1c) level, and tinea pedis diagnosis. HbA1c values before the diagnosis of tinea pedis were used to assess glycemic control in diabetic patients at the time of tinea pedis diagnosis. HbA1c% values of <7, 7.0–7.9, and ≥8 were considered as recommended control, less-stringent control, and poor control of diabetes mellitus, respectively. All data were fully anonymized, with no personal information accessible to the research team.

### Statistical analysis

Statistical analysis was performed using the Statistical Package for the Social Sciences (SPSS) version 28 in association with Microsoft Excel. Descriptive statistics summarized the data, and associations between tinea pedis and risk factors were assessed. The nationality classification followed the United Nations’ classification system, and the “other-miscellaneous group” included individuals whose nationalities were not well-represented in the dataset (specifically those with nationalities having small sample sizes ≤1%). The “other-miscellaneous group” was selected as the reference category for analyzing nationality-related risk factors. Heterogeneity of the “other-miscellaneous group” provides a neutral comparator, avoiding inherent bias and enabling unbiased comparisons across the more distinct homogeneous nationality groups (e.g., Qatari or Southeast Asian) as the reference, which may have distinct socioeconomic, cultural, or health characteristics. Also, it is practical in multivariate analysis, ensuring robust statistical modeling without overrepresentation or underrepresentation.

A *p*-value of <0.05 was considered statistically significant. To measure the strength of association between a dichotomous independent variable (such as a specific group compared to a reference group) and a dichotomous outcome variable (like diagnosed with tinea pedis), the prevalence ratio (PR) was used. The logarithm method was used in calculating confidence intervals for PR. A multiple logistic regression model with selected factors as independent variables and having tinea pedis as the dependent variable was used. The model assessed the risk of having tinea pedis for each explanatory variable, adjusting for the effect of other confounders included in the model. We obtained approval with reference number BUHOOTH-D-23-00039 to conduct this study from the Institutional Review Board (IRB) at PHCC, and we were exempt from obtaining patient consent.

## Results

The results presented were based on the analysis of 1,002,594 EMRs of adults registered with PHCC. As shown in Table 1, the study sample consisted of the highest proportion of adults aged 30–49 years (58.7%), while older adults (≥70 years) constituted only 1.9% of the sample. Males were slightly over half (51%) of the studied sample. The most frequent nationality group was Southeast Asian (32.7%), followed by locals (Qatari, 19.5%).

As shown in Table 2, the overall prevalence of tinea pedis among the adult population was 1.8%. The prevalence among diabetic patients was 6.8%, compared to 1% among those without diabetes. Being diabetic significantly increases the risk of having tinea pedis by 7.1 times, as illustrated in Table 2.

As shown in Table 3, the risk of having tinea pedis increases significantly with advancing age. The prevalence rate increases by 25.1 times in older ages (≥70 years) compared to the youngest adults (18–29 years). Male gender is associated with a significant increase of 1.6 times in the risk of being diagnosed with tinea pedis compared to females. Among nationality groups, the Southeast Asian group had the lowest prevalence of tinea pedis (0.2%). Compared to the reference category (the other-miscellaneous group), the risk was reduced by 5 times (PR 0.2) in the Southeast Asia nationality group. Conversely, the remaining nationality groups were associated with an increase in risk, which can reach as high as 3.9 times among the North African nationality group. The risk of being diagnosed with tinea pedis is positively associated with obesity. An increase in BMI is associated with a progressive increase in risk of reaching the highest increase of 15.1 times among the morbid obesity group.

As shown in Table 4, less stringent control and poor control of HbA1c are associated with a significant increase of 30% and 20%, respectively, in the risk of being diagnosed with tinea pedis compared to the recommended control of HbA1c. The risk showed a statistically significant positive trend with aging and increasing BMI. Furthermore, male gender increased the risk of tinea pedis by 10% compared to females. Moreover, the following nationality groups: Qatar, North Africa, Sub-Saharan Africa, North America/Europe/Australasia, and West Asia were associated with a significant increase in risk compared to the reference nationality category (other, miscellaneous), while the Southeast Asia and South Asia groups were associated with a risk reduction.

The previously studied explanatory variables were tested in a multivariate model to assess the net and independent contributions of each of them to the overall risk of being diagnosed with tinea pedis. The model was statistically significant and accurately predicted tinea pedis classification with a 91.2% accuracy rate. Aging was the strongest predictor. Compared to those younger than 30 years, the risk increased 3.2 times among those 30–49 years old, reaching a maximum increase of 12.5 times in those ≥70 years after controlling for the remaining confounders included in the model. Male gender increased the risk of being diagnosed with tinea pedis by 40% after controlling for the remaining confounders included in the model. Diabetics with uncontrolled diabetes (HbA1c ≥ 7) had a similar increased risk by 40%. Compared to non-obese, the risk was 1.3 times among obese grade-I, and 2.1 times among morbidly obese individuals after controlling for the remaining confounders included in the model. Apart from the Southeast Asia nationality group, all the remaining groups were associated with risk ranging between 1.1 times (in South Asia) and as high as 2.2 times (in North Africa), as shown in Table 5.

## Discussion

In this study, participants were included from several nationalities, with 19.5% being Qatari nationals. The current study found an overall prevalence of tinea pedis of 1.8%. When compared to the worldwide prevalence of 3%,^2^ the prevalence in Qatar is relatively low. This difference could be attributed to several factors, including geographic location, climate variations, and healthcare practices. It’s also crucial to consider variations within specific population subgroups.

The risk of having tinea pedis increased significantly with advancing age, with individuals aged 70 years or older being 25 times more likely to have the condition compared to those aged 18–29 years. According to the analysis of the United States database,^14^ the prevalence of tinea pedis in individuals aged 75 years or older was shown to be higher than in younger individuals. The multivariate analysis in the current study showed that individuals aged 75 years or older had 1.45 times higher risk of tinea pedis compared to younger individuals. Several factors may contribute to this association, including inactivity, challenges with foot care, and exposure to contaminated environments in nursing homes. The same factors that contribute to an elevated risk of tinea pedis in older adults might also explain the higher odds of the condition in individuals with physical disabilities. Findings regarding age-specific prevalence are consistent with previous epidemiological studies.^14^ This consistency supports the reliability of our study for investigating the burden of skin fungal infections, particularly in the elderly group.

Males represented 51% of the studied sample. Qatar’s population has a significant male majority (71% male),^15^ primarily due to the large number of male expatriate workers in industries such as construction. Many male expatriate workers are provided with employer-provided private healthcare or are served by the Red Crescent Society. In this study, we noted a significant increase in the risk of having tinea pedis in males compared to females. Males were 1.6 times more likely to be diagnosed with tinea pedis compared to females. In a study conducted in Madrid, Spain, the prevalence of tinea pedis was 4.2% for males and 1.7% for females. This difference persisted in the multivariate analysis, even after controlling for other factors, with males having a 2.6 times higher risk for tinea pedis than females. The increased prevalence in men may be due to a higher incidence of trauma to the nails and more frequent use of occlusive footwear.^4^

Among the nationality groups in the current study, the highest prevalence of tinea pedis was found among North Africans (3%). A study conducted in Tunisia reported that a sizable proportion of participants engaged in ritual washing. The religious practice of doing five daily ablutions can lead to foot maceration and increase the risk of fungal penetration through the stratum corneum of the epidermis. It may be linked to the growth of fungi in places where people wash and on the prayer carpets. Furthermore, 50.5% of foot mycosis patients use public restrooms for bathing and showering. This high frequency could also be attributed to Tunisians’ custom and practice of frequenting hammams, which are warm, humid places that serve as sources of fungal contagion.^16^

A positive association was observed between BMI and tinea pedis risk in the current study. Morbidly obese individuals were at the highest risk, being 15 times more likely to have tinea pedis compared to underweight individuals. This has been demonstrated in multiple previous studies for multiple reasons. According to a nationwide study among Korean adults, obesity was a significant risk factor. People with obesity often have thicker subcutaneous fat layers and deeper skin folds. This can trap moisture and warmth, creating an ideal environment for fungal growth, including the dermatophytes that cause conditions like tinea pedis.^8^ Moreover, obesity is linked to conditions such as diabetes mellitus, which can further elevate the risk.

Out of 100 persons with diabetes, seven have tinea pedis. Diabetic individuals are 7.1 times more likely to have tinea pedis compared to non-diabetic individuals. A large-scale database study conducted in Japan found that diabetes is an independent risk factor for developing tinea pedis. This is likely because diabetes can reduce the body’s resistance to fungal infections and lead to poor circulation in the feet, which can impair wound healing and increase the likelihood of infections. Additionally, diabetes can lead to nerve damage in the feet, known as diabetic peripheral neuropathy, which can impair a person’s ability to detect injuries or infections.^17^ Our study also examined risk factors among diabetics and found that inadequately or poorly controlled hyperglycemia significantly increased the risk of tinea pedis. People with higher blood glucose levels are more prone to fungal infections.^18^

The preventive measures for tinea pedis include maintaining good foot hygiene, keeping feet dry, especially between toes, wearing well-ventilated shoes, avoiding walking barefoot in public spaces like locker rooms and swimming pools, using moisture-wicking socks and changing them frequently, especially after sweating, and avoiding sharing personal items like towels.^10^ Topical antifungal therapy, typically applied once or twice daily for 1–6 weeks, is the primary treatment for superficial or localized tinea pedis. Common topical antifungal agents include allylamines (e.g., terbinafine), azoles (e.g., ketoconazole), benzylamine, ciclopirox, tolnaftate, and amorolfine. For more severe cases, oral antifungal agents such as terbinafine, itraconazole, and fluconazole may be used. Combining topical and oral antifungal treatments can improve the cure rate. With appropriate treatment, the prognosis is generally favorable; however, if left untreated, the lesions may persist and worsen.^3^

## Strengths and Limitations

This study included a large and representative sample size of over 1 million EMR and involved approximately one-third of the Qatar population.^15^ This substantial sample size and real-world data enhance the study’s statistical power, allowing for greater generalizability of the findings to the adult population of Qatar. Detailed demographic and clinical information, along with comprehensive statistical analysis, investigated the relationships between tinea pedis and various risk factors. Findings of this study were compared with those of previous studies conducted in other countries, providing valuable context for understanding the observed prevalence rates and risk factors. Despite these strengths, this study may have limitations. EMR may not capture all cases of tinea pedis, and data may have limitations related to diagnostic accuracy. Also, knowing whether these patients with tinea pedis were wearing closed or open shoes could have provided valuable information.

## Conclusion

This study is the first of its kind in Qatar, providing valuable information about the prevalence and risk factors of tinea pedis. The prevalence of tinea pedis was 1.8%, and there was a consistent association of tinea pedis and risk factors like advancing age, male gender, obesity, being diabetic, and poorly controlled diabetes. These results can help raise awareness among local physicians to screen certain high-risk groups and further promote prevention by encouraging patients to practice proper foot hygiene. Better control of diabetes would reduce the risk of having this dermatologic fungal infection, according to our study.

## List of abbreviations

EMR Electronic medical records

PHCC Primary Health Care Corporation

BMI Body mass index

HbA1c Hemoglobin A1c

## Ethical approval

We received an approval notice with reference number BUHOOTH-D-23-00039 to conduct this research from the Institutional Review Board at Primary Health Care Corporation in Qatar.

## Acknowledgements

The publication of this article was funded by the Primary Health Care Corporation in Qatar.

## Conflicts of interest

All authors declare no conflicts of interest.

## Data availability

Data are available on reasonable request from the corresponding author.

## Figures and Tables

**Table 1.** Frequency distribution of the study sample by sociodemographic variables.

	***n***	**%**
**Age group (years)**		
18–29	226,297	22.6
30–49	588,429	58.7
50–69	169,274	16.9
≥70	18,594	1.9
Total	1,002,594	100.0
**Gender**		
Female	491,383	49.0
Male	511,211	51.0
Total	1,002,594	100.0
**Nationality groups**		
South Asia	327,396	32.7
Qatar	195,063	19.5
Southeast Asia	120,809	12.0
North Africa	104,698	10.4
North America/Europe/Australasia	97,923	9.8
West Asia	93,106	9.3
Sub-Saharan Africa	58,186	5.8
Other (miscellaneous)/unknown regions	5413	0.5
**Body mass index categories**		
Underweight (<18.5)	14,022	2.5
Acceptable (18.5–24.9)	146,842	26.6
Overweight (25–29.9)	197,089	35.8
Obese grade I (30–34.9)	120,379	21.8
Obese grade II (35–39.9)	47,959	8.7
Morbid obesity (≥40)	24,803	4.5
**Total**	551,094	100.0

**Table 2.** Prevalence and diabetes risk of being diagnosed with tinea pedis among the adult population in PHCC in Qatar.

	**Tinea pedis**					
	**Negative**		**Positive**		**Total**	
	***n***	**%**	***n***	**%**	***n***	**%**	**PR**	**95% CI**	***p***
**DM**									
Negative	857,942	99.0	8385	1.0	866,327	100.0	Ref
Positive	126,955	93.2	9312	6.8	136,267	100.0	7.1	(6.86–7.27)	<0.001
Total	984,897	98.2	17697	1.8	1,002,594	100.0

DM: diabetes mellitus, PR: prevalence Ratio, CI: confidence interval.

**Table 3.** Relative frequency of tinea pedis among the adult population in PHCC in Qatar by selected explanatory variables.

	**Tinea pedis**					
	**Negative**		**Positive**		**Total**	
	***n***	**%**	***n***	**%**	***n***	**%**	**PR**	**95% CI**	***p***
**Age group (years)**									
18–29	225,198	99.5	1099	0.5	226,297	100.0	Ref		
30–49	581,345	98.8	7084	1.2	588,429	100.0	2.5	(2.33–2.64)	<0.001
50–69	162,023	95.7	7251	4.3	169,274	100.0	8.8	(8.28–9.39)	<0.001
≥70	16,331	87.8	2263	12.2	18,594	100.0	25.1	(23.35–26.89)	<0.001
Total	984,897	98.2	17,697	1.8	1,002,594	100.0			
**Gender**									
Female	484,718	98.6	6665	1.4	491,383	100.0	Ref		
Male	500,179	97.8	11,032	2.2	511,211	100.0	1.6	(1.54–1.64)	<0.001
**Nationality groups**									
Other (miscellaneous)/unknown regions	5372	99.2	41	0.8	5413	100.0	Ref		
Qatar	190,312	97.6	4751	2.4	195,063	100.0	3.2	(2.37–4.37)	<0.001
North Africa	101,601	97.0	3097	3.0	104,698	100.0	3.9	(2.88–5.31)	<0.001
Sub-Saharan Africa	57,127	98.2	1059	1.8	58,186	100.0	2.4	(1.76–3.27)	<0.001
North America/Europe/Australasia	96,008	98.0	1915	2.0	97,923	100.0	2.6	(1.9–3.51)	<0.001
Southeast Asia	120,613	99.8	196	0.2	120,809	100.0	0.2	(0.15–0.29)	<0.001
South Asia	322,753	98.6	4643	1.4	327,396	100.0	1.9	(1.38–2.54)	<0.001
West Asia	91,111	97.9	1995	2.1	93,106	100.0	2.8	(2.08–3.85)	<0.001
**BMI categories**									
Underweight (<18.5)	13,966	99.6	56	0.4	14,022	100.0	Ref
Acceptable (18.5–24.9)	144,951	98.7	1891	1.3	146,842	100.0	3.2	(2.47–4.2)	<0.001
Overweight (25–29.9)	192,132	97.5	4957	2.5	197,089	100.0	6.3	(4.84–8.19)	<0.001
Obese grade I (30–34.9)	116,167	96.5	4212	3.5	120,379	100.0	8.8	(6.73–11.4)	<0.001
Obese grade II (35–39.9)	45,801	95.5	2158	4.5	47,959	100.0	11.3	(8.65–14.68)	<0.001
Morbid obesity (≥40)	23,307	94.0	1496	6.0	24,803	100.0	15.1	(11.57–19.7)	<0.001

BMI: body mass index, PR: prevalence ratio, CI: confidence interval.

**Table 4.** Relative frequency of tinea pedis among diabetic adults in PHCC in Qatar by selected explanatory variables.

	**Tinea pedis**					
	**Negative**		**Positive**		**Total**	
	***n***	**%**	***n***	**%**	***n***	**%**	**PR**	**95% CI**	***p***
**HbA1c (%)**									
Recommended control (HbA1c < 7)	51,491	93.0	3891	7.0	55,382	100	Ref		
Less-stringent control (HbA1c 7.0–7.9)	20,638	90.8	2094	9.2	22,732	100	1.3	(1.25–1.38)	<0.001
Poor control (HbA1c ≥ 8)	30,428	91.7	2743	8.3	33,171	100	1.2	(1.14–1.25)	<0.001
**Age group (years)**									
18–29	3868	98.6	54	1.4	3922	100.0	Ref		
30–49	50,530	96.1	2038	3.9	52,568	100.0	2.8	(2.16–3.69)	<0.001
50–69	61,622	92.2	5230	7.8	66,852	100.0	5.7	(4.35–7.41)	<0.001
≥70	10,935	84.6	1990	15.4	12,925	100.0	11.2	(8.55–14.62)	<0.001
Total	126,955	93.2	9312	6.8	136,267	100.0			
Gender									
Female	53,308	93.6	3659	6.4	56,967	100.0	Ref		
Male	73,647	92.9	5653	7.1	79,300	100.0	1.1	(1.07–1.16)	<0.001
**Nationality groups**									
Other (miscellaneous)/unknown regions	175	94.6	10	5.4	185	100.0	Ref		
Qatar	35,579	91.5	3295	8.5	38,874	100.0	1.6	(0.86–2.87)	0.52
North Africa	11,138	89.3	1332	10.7	12,470	100.0	2.0	(1.08–3.63)	0.15
Sub-Saharan Africa	3728	91.4	350	8.6	4078	100.0	1.6	(0.86–2.93)	0.51
North America/Europe/Australasia	11,878	92.6	943	7.4	12,821	100.0	1.4	(0.74–2.49)	0.8
Southeast Asia	7258	99.2	61	0.8	7319	100.0	0.2	(0.08–0.29)	<0.001
South Asia	46,547	95.4	2260	4.6	48,807	100.0	0.9	(0.47–1.57)	0.97
West Asia	10,652	90.9	1061	9.1	11,713	100.0	1.7	(0.92–3.08)	0.4
**BMI categories**									
Underweight (<18.5)	388	97.5	10	2.5	398	100.0	Ref		
Acceptable (18.5–24.9)	14,902	94.6	844	5.4	15,746	100.0	2.1	(1.15–3.94)	0.1
Overweight (25–29.9)	33,589	93.1	2495	6.9	36,084	100.0	2.8	(1.49–5.08)	0.008
Obese grade I (30–34.9)	26,571	91.5	2480	8.5	29,051	100.0	3.4	(1.84–6.28)	<0.001
Obese grade II (35–39.9)	12,818	90.1	1402	9.9	14,220	100.0	3.9	(2.12–7.24)	<0.001
Morbid obesity (≥40)	7671	87.9	1057	12.1	8728	100.0	4.8	(2.61–8.91)	<0.001

BMI: body mass index, PR: prevalence ratio, CI: confidence interval.

**Table 5.** Multiple logistic regression modeling the risk of being diagnosed with tinea pedis among diabetic adults in PHCC in Qatar by selected explanatory variables.

	**Partial OR**	**95% CI OR**	***p***
**Age group (years)**			
(30–49 years) compared to younger adults (18–29 years)	3.2	(2.4–4.4)	<0.001
(50–69 years) compared to younger adults (18–29 years)	6.5	(4.8–8.8)	<0.001
(≥70 years) compared to younger adults (18–29 years)	12.5	(9.1–17)	<0.001
**Males compared to females**	1.4	(1.4–1.5)	<0.001
**Uncontrolled (HbA1c ≥ 7) compared to the recommended control**	1.4	(1.3–1.4)	<0.001
**BMI categories**			
Obese grade I compared to non-obese (<30 kg/m2)	1.3	(1.3–1.4)	<0.001
Obese grade II compared to non-obese (<30 kg/m2)	1.6	(1.5–1.7)	<0.001
Obese grade III compared to non-obese (<30 kg/m2)	2.1	(2–2.3)	<0.001
**Nationality groups**			
North Africa compared to other (miscellaneous)/unknown regions	2.2	(1–4.8)	0.044
Sub-Saharan Africa compared to other (miscellaneous)/unknown regions	1.8	(0.8–3.9)	0.14
North America/Europe/Australasia compared to other (miscellaneous)/unknown regions	1.6	(0.7–3.5)	0.23
West Asia compared to other (miscellaneous)/unknown regions	1.5	(0.7–3.3)	0.28
Qatar compared to other (miscellaneous)/unknown regions	1.3	(0.6–2.9)	0.47
South Asia compared to other (miscellaneous)/unknown regions	1.1	(0.5–2.3)	0.86
Southeast Asia compared to other (miscellaneous)/unknown regions	0.2	(0.1–0.5)	<0.001

Overall classification accuracy = 91.2%. *p* (Model) <0.001. BMI: body mass index, OD: odd ratio, CI: confidence interval.
